# Flexible Composite Hydrogels Based on Polybenzoxazine for Supercapacitor Applications

**DOI:** 10.3390/gels10030197

**Published:** 2024-03-13

**Authors:** Shakila Parveen Asrafali, Thirukumaran Periyasamy, Gazi A. K. M. Rafiqul Bari, Seong-Cheol Kim

**Affiliations:** 1 School of Chemical Engineering, Yeungnam University, 280 Daehak-ro, Gyeongbuk, Gyeongsan 38541, Republic of Korea; shakilaparveen@yu.ac.kr; 2 Department of Fiber System Engineering, Yeungnam University, 280 Daehak-ro, Gyeongbuk, Gyeongsan 38541, Republic of Korea; thirukumaran@ynu.ac.kr; 3 School of Mechanical Smart and Industrial Engineering, Gachon University, 1342 Seongnam-daero, Sujeong-gu, Seongnam-si 13120, Republic of Korea

**Keywords:** polybenzoxazine, PVA, composite films, flexible electrolyte, supercapacitor, hydrogels

## Abstract

Polybenzoxazines (Pbzs) are advanced forms of phenolic resins that possess many attractive properties, including thermal-induced self-curing polymerization, void-free polymeric products and absence of by-product formation. They also possess high T_g_ (glass transition temperature) and thermal stability. But the produced materials are brittle in nature. In this paper, we present our attempt to decrease the brittleness of Pbz by blending it with polyvinylalcohol (PVA). Benzoxazine monomer (Eu-Ed-Bzo) was synthesized by following a simple Mannich condensation reaction. The formation of a benzoxazine ring was confirmed by FT-IR and NMR spectroscopic analyses. The synthesized benzoxazine monomer was blended with PVA in order to produce composite films, PVA/Pbz, by varying the amount of benzoxazine monomer (1, 3 and 5 wt. % of PVA). The property of the composite films was studied using various characterization techniques, including DSC, TGA, water contact angle analysis (WCA) and SEM. WCA analysis proved that the hydrophobic nature of Pbz (value) was transformed to hydrophilic (WCA of PVA/Pbz5 is 35.5°). These composite films could play the same role as flexible electrolytes in supercapacitor applications. For this purpose, the composite films were immersed in a 1 M KOH solution for 12 h in order to analyze their swelling properties. Moreover, by using this swelled gel, a symmetric supercapacitor, AC//PVA/Pbz5//AC, was constructed, exhibiting a specific capacitance of 170 F g^−1^.

## 1. Introduction

In the last few decades, multicomponent thermosets have gained considerable interest as they could improve the properties of a polymer by preparing blends or composites. Elastomers and thermoplastics have been incorporated into thermosetting polymers (i.e., epoxy resins) to reduce their shrinkage and improve their thermo-mechanical properties. However, the miscibility of the polymer blend does not occur easily at the molecular level. With the existence of favorable intermolecular interactions, such as hydrogen bonding, the miscibility of a polymer with an additional functional group could favor polymer miscibility and result in the formation of homogenous blends [1,2,3,4,5,6,7,8,9].

Polybenzoxazine (PBz) is an attractive class of phenolic resin that possesses an excellent balance of mechanical, physical and thermal properties, along with a wide range of molecular design flexibility. Benzoxazine monomers can be synthesized from inexpensive raw materials through Mannich condensation. Additionally, they can be cured without any catalyst and do not produce any by-products during polymerization [10,11,12,13,14]. Polymerization of benzoxazine monomers proceeds via a heterocyclic ring-opening process, producing linear or cross-linked structures, depending on benzoxazine functionality. Polybenzoxazine possesses good dimensional stability due to its near-zero volumetric shrinkage and has excellent processability due to wide range of molecular design flexibility. Pbz can withstand higher temperatures without degradation, which makes it suitable for high-temperature applications. During polymerization, it exhibits low shrinkage and maintains its dimensional stability, thus making the manufacturing process more advantageous. Pbzs also exhibit excellent flame-retardant properties, and so, they can be used as electrical insulators. They also possess very low dielectric constants and can be used as electronic components. The commercially available polybenzoxazine [bisphenol-A-based Pbz] is brittle in nature due to its aromatic groups. Due to its brittle nature, processing Pbz into different forms, such as films, is not possible. However, by making use of the molecular design flexibility, benzoxazines with the required functional groups can be synthesized to produce their different forms. The potential advantages of Pbz can be utilized by improving its toughness [15,16,17,18,19]. The performance of polybenzoxazine can be improved by two different approaches: one is to synthesize novel benzoxazines by modifying their structure, and the other is to prepare blends or composites with other polymers or inorganic fillers [20,21,22,23].

Flexible solid-state supercapacitors (FSSCs) have gained attention in recent years due to their improved properties, including long-term cyclic stability, high power density and fast charge–discharge rate. Due to these enhanced properties, these FSSCs find their use in wearable and portable electronic devices. Modifications in FSSCs can be brought about by producing novel electrode materials and electrolytes. Porous carbon materials, including activated carbon and graphene, metal oxides and conductive polymers, can be used as electrode materials. And in the case of electrolytes, solid electrolytes, liquid state electrolytes and gel electrolytes can be used. Generally, polymer gel electrolytes, consisting of conductive polymers like polyvinyl alcohol (PVA), poly(ethylene oxide) (PEO), polyacrylamide (PAM), polyacrylic acid (PAA) and poly(methylmethacrylate) (PMMA), are used in flexible supercapacitors due to their highly cross-linked network [24,25,26,27]. Moreover, these electrolytes provide easy processability, possess high flexibility and dimensional stability, enlarge the potential window and are eco-friendly in nature. Among these polymers, PVA-based gel electrolytes are extensively studied, as they possess extreme hydrophilicity due to the presence of hydroxyl (–OH) groups. In addition, this –OH functionality of PVA can bind with water molecules and other compounds through hydrogen-bonding interactions [28,29,30,31].

Aziz et al. [26] prepared plasticized PVA-based polymer electrolytes using a solution cast method and studied the effect of glycerol concentration on the PVA-KI electrolyte. The work proved that PVKI4 (PVA:KI with 40 wt. % of glycerol) displayed a specific capacitance of 152.4 F g^−1^ at 1 A g^−1^. In another work, Deng et al. [25] synthesized an alkaline gel electrolyte containing redox-active PVA-KOH-urea-LiClO_4_. An asymmetric supercapacitor was assembled using this alkaline gel electrolyte, where Co oxide or Mn oxide were used as electrodes. This asymmetric supercapacitor exhibits enhanced specific capacitance of 186 F g^−1^ at a higher current density of 2 A g^−1^. Moreover, it also showed a capacitance retention of 89.5% after 6000 cycles. In a similar way, Lin et al. [24] used a PVA-based gel electrolyte and fabricated a symmetric supercapacitor with a Zn-NC electrode (nitrogen-doped carbon on zinc particles). The work showed that the symmetric cells could maintain cyclic stability for a period of 3500 h @ 1 mA cm^–2^ and a period of 850 h @ 5 mA cm^–2^. Wang et al. [32] prepared a composite material with a multi-layered hierarchical structure containing cellulose nanofibers/carbon and nanotubes/vinasse-activated carbon [CNFs/CNTs/VAC] by following a simple vacuum filtration and freeze-drying method. The composite material exhibited excellent integration performance, stable energy storage performance and EMI shielding performance.

Taking into account the ideas obtained from previous works, we fabricated a novel polymer gel electrolyte using PVA and Pbz. A Eugenol and ethylene diamine-based benzoxazine monomer was synthesized following a Mannich condensation reaction. The monomer undergoes self-polymerization in the presence of temperature to form Pbz-containing hydroxyl and amine groups, which blend well with PVA for the formation PVA/Pbz films. The weight % of Pbz was varied between 1 and 5% to avoid any agglomeration. The hydrogen-bonding interactions between –OH groups of PVA and –OH and –NH_2_ groups of Pbz result in stable composite PVA/Pbz hydrogels. The ethylene diamine linkage in the benzoxazine monomer adds flexibility, and the amine and hydroxyl groups of Pbz allow it to blend with PVA to form a stable film. Additionally, a symmetric supercapacitor was fabricated using this composite hydrogel as an electrolyte and activated carbon as an electrode, and its electrochemical measurement was analyzed and discussed in detail.

## 2. Materials and Methods

### 2.1. Materials

Polyvinyl alcohol (PVA, Average mol. wt. = 89,000–98,000) (341584), eugenol (8.18455), ethylene diamine (8.00947), p-formaldehyde (158127) and dimethyl sulfoxide (472301) were purchased from Sigma Aldrich Korea, Gangnam-Gu 06178, Republic of Korea. Potassium hydroxide (1310-58-3), NMP (872-50-4), PVDF (24937-79-9) and activated carbon (7440-44-0) were purchased from DAESUNG662 Kyunginro, Guro-gu, Seoul, Republic of Korea. All chemicals and solvents were used without further purification.

### 2.2. Characterization

Fourier transform infrared (FT-IR) spectra were obtained with a Perkin Elmer MB3000 FT-IR spectrometer. The spectra were obtained at a resolution of 4 cm^−1^ in the IR range of 4000–400 cm^−1^. Samples were prepared by grinding with KBr and compressed to form discs. Nuclear magnetic resonance (NMR) spectra were recorded by using an Agilent NMR, VNS600 at a proton frequency of 600 MHz for ^1^H-NMR. Solutions were prepared by dissolving the samples in DMSO-d_6_. Differential scanning calorimetric (DSC) analysis was carried out using a TA Instruments, Q200 model at a heating rate of 10 °C/min and with a nitrogen flow rate of 50 mL/min. Samples weighing between 5 and 9 mg were crimped in hermetic aluminium pans with lids and used for analysis. Thermogravimetric analysis (TGA) was carried out using a TA Instruments, SDT Q600 model at a heating rate of 10 °C/min up to 800 °C under N_2_ atmosphere. Morphological analyses were performed on a field emission scanning electron microscopy (FESEM, Hitachi S-4800, Tokyo, Japan) at an accelerating voltage of 10 kV. A Dataphysics Instrument OCA 20 model was used to determine the water contact angle of the PVA and PVA/Pbz films. The film was placed horizontally on the glass slide and 2 µL of DI water was dropped onto the film using a micro syringe. The measuring range of the instrument is 0 to 180°. For each sample, the contact angle measurement was taken in five different areas and the average value is denoted.

### 2.3. Electrochemical Measurments

All electrochemical measurements, including cyclic voltammetry (CV), galvanostatic charge–discharge (GCD) and electrochemical impedance spectroscopy (EIS), were performed on the CorrTest-CS350 electrochemical workstation. The CV measurements were carried out at a potential window from −1.0 and 0.0 V under the different scan rates from 5 to 100 mV s^−1^. The GCD measurements were performed with a potential window of −1.0 and 0.0 V at the current densities, and they varied from 0.5 to 2.5 A g^−1^. EIS measurements were performed in the frequency range of 0.01 kHz–100 kHz with an alternating current amplitude of 5 mV. All the electrochemical tests were conducted at room temperature. The capacitances of the electroactive materials were obtained from their GCD curves according to the following equation [2].

*C_s_* = *I* ∆*t*/*m* ∆*V* (1)

where *C_s_* is the specific capacitances (F g^−1^), *I* is the current in the charge–discharge process (A), Δ*t* represents to the discharge time (s), Δ*V* stands for the potential window during the charge–discharge measurement (V) and *m* donates the mass of the electroactive materials (g).

### 2.4. Synthesis of Benzoxazine Monomer (6-Allyl-8-methoxy-3-ethylamine-3,4-dihydro-1,3-benzoxazine) (Eu-Ed-Bzo)

**Scheme 1:**
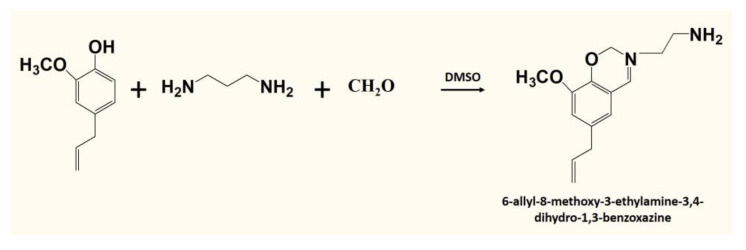
Synthesis of benzoxazine monomer (Eu-Ed-Bzo).

At first, paraformaldehyde (1.8 g, 0.06 m) and DMSO (20 mL) was stirred at 50 °C. After obtaining a clear solution, ethylene diamine (1.34 mL, 0.02 m) was added dropwise, followed by the addition of eugenol (3.28 g, 0.02 m). Finally, the temperature was raised to 120 °C and the reaction was carried forward for 3 h. The yellow-colored solution obtained after the completion of the reaction was precipitated in NaOH solution (1 N). After several washings with DI water, the product was finally dried in a vacuum at 50 °C for 12 h to obtain the Eu-Ed-Bzo monomer (Scheme 1).

### 2.5. Preparation of PVA/Pbz Composite Films

**Scheme 2:**
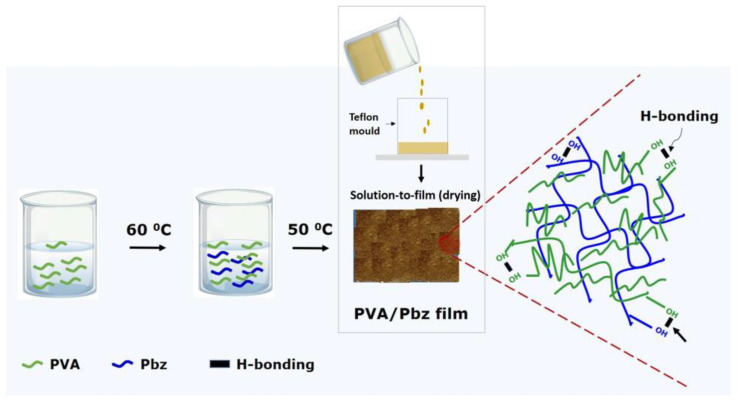
Preparation of PVA/Pbz composite films.

To prepare the composite films, at first, the PVA solution (10 wt. %) was stirred overnight at 60 °C to completely dissolve the PVA. Then, to this PVA solution, different amounts of Eu-Ed-Bzo were added (1, 3 and 5 wt. % with respect to PVA solution). This solution was then allowed to stir continuously for 5 h, maintaining the temperature at 100 °C. After which, the mixed solution was transferred to a teflon mold and, after drying at 50 °C, free standing films were produced, denoted as PVA/Pbz1, PVA/Pbz3 and PVA/Pbz5, respectively. In a similar way, PVA films, containing 10 wt. % of PVA, was also produced. All these films were characterized for several analyses (Scheme 2).

## 3. Results and Discussion

### 3.1. Structure Analysis of Eu-Ed-Bzo

**Figure 1:**
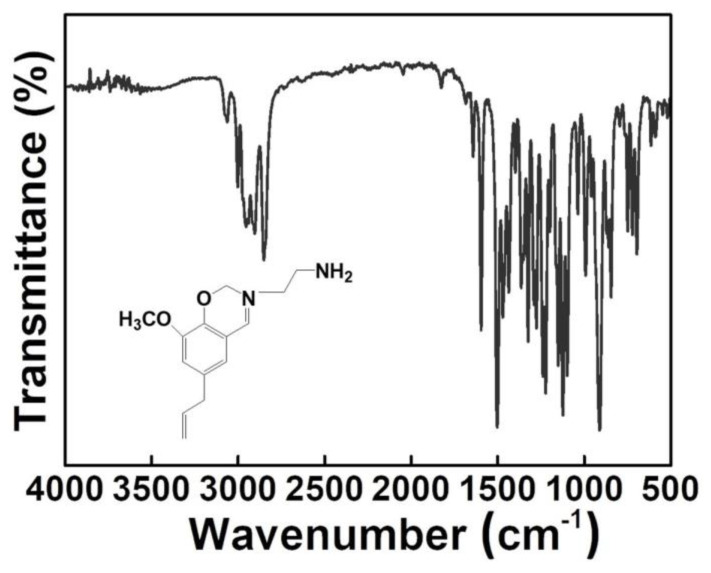
FT-IR spectrum of Eu-Ed-Bzo.

The FT-IR spectrum of the benzoxazine monomer was represented in Figure 1. The formation of benzoxazine ring was observed via the vibrations of –CH_2_, C–O–C and C–N–C in the oxazine ring. The bands corresponding to 990 cm^−1^, 1236 and 1218 cm^−1^, and 1120 and 1094 cm^−1^ prove the formation of the benzoxazine ring structure. Other vibrations due to the methoxy group, tetra substituted benzene, aliphatic and aromatic C–H stretching were found at 1272, 1360, 2850 and 2906–3076 cm^−1^, respectively.

**Figure 2:**
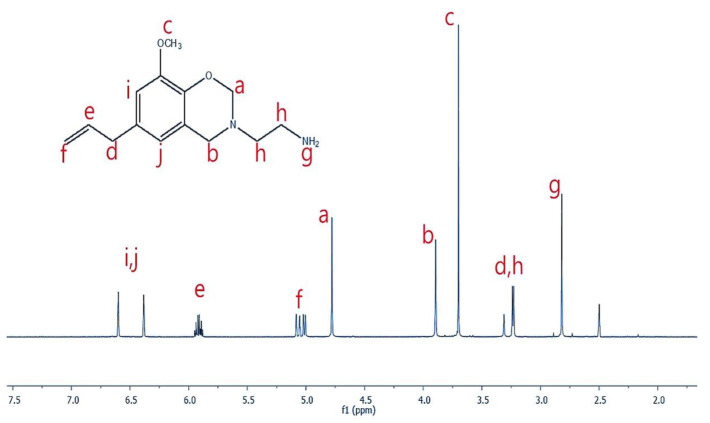
^1^H-NMR spectrum of Eu-Ed-Bzo.

Figure 2 represents the ^1^H-NMR spectrum of the benzoxazine monomer. The oxazine ring protons gave two distinct singlets at 4.7 ppm (O–CH_2_–N) and 3.8 ppm (N–CH_2_–Ar). This further confirms the successful formation of benzoxazine ring structure. The other proton signals were found at 2.8 ppm (amine protons), 3.7 ppm (methoxy protons), 3.2 ppm (ethyl amine protons), 5.0 and 5.9 ppm (allyl protons) and 6.3 and 6.6 ppm (aromatic protons). Thus, the structure of the benzoxazine monomer is confirmed by FT-IR and NMR spectroscopic analyses.

### 3.2. DSC Analysis

**Figure 3:**
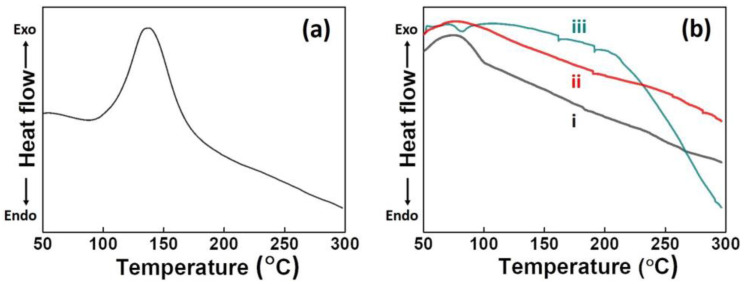
DSC profile of (**a**) Eu-Ed-Bzo, and (**b**) Eu-Ed-Bzo/PVA films [(i) PVA/Pbz1; (ii) PVA/Pbz3; and (iii) PVA/Pbz5].

Figure 3a,b represents the curing behavior of Eu-Ed-Bzo and PVA/Pbz composite films. The DSC curve of the benzoxazine monomer, Eu-Ed-Bzo (Figure 3a), displays a single exothermic peak. This exotherm shows the curing process with an onset curing temperature at 91 °C, maximum curing temperature at 133 °C and final curing temperature at 178 °C. In general terms, the bisphenol-A-based benzoxazine monomer, BA-a, shows a curing profile above 200 °C. But, the eugenol-ethylenediamine-based benzoxazine monomer, Eu-Ed-Bzo, due to the presence of allyl, methoxy and amine group, reduces the curing profile. This, in turn, makes the processability of the Bzo monomer very easy. The PVA/Pbz composite films do not show any curing profile (Figure 3b), which means that, during the blending process, a temperature of 100 °C is sufficient to cure the benzoxazine monomer and to form the composite films. Moreover, a slight hump in the curve at 170 °C could be observed, indicating the glass transition temperature (T_g_) [32,33].

### 3.3. Water Contact Angle Analysis

**Figure 4:**
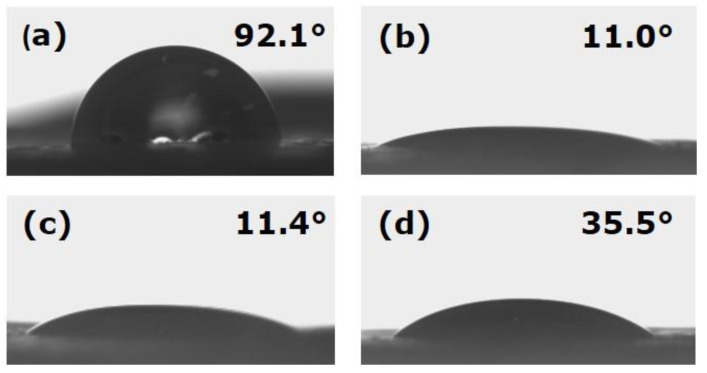
Water contact angle images of (**a**) PVA, (**b**) PVA/Pbz1, (**c**) PVA/Pbz3, and (**d**) PVA/Pbz5.

Water contact angle analysis (WCA) is a surface analysis and is used to determine the hydrophilic or hydrophobic nature of the materials. Generally, based on the contact angle formed by the water droplet on the surface of the material, the material is denoted as superhydrophilic (θ = <10°), hydrophilic (θ = 10°–90°), hydrophobic (θ = 90°–120°) and superhydrophobic (θ = >120°). Each of the material properties finds an application in respective fields. Figure 4 displays the WCA images of Pbz and PVA/Pbz composite films. The neat Pbz is hydrophobic in nature with a contact angle of 92.1°. As polyvinylalcohol is a hydrophilic polymer, the PVA/Pbz composite films showed a hydrophilic nature. The WCA of PVA/Pbz1 and PVA/Pbz3 showed the lowest contact angle of 11.0° and 11.4°, whereas PVA/Pbz5 showed a slightly higher contact angle of 35.5° due to the increased weight content of the benzoxazine monomer.

### 3.4. SEM Analysis

**Figure 5:**
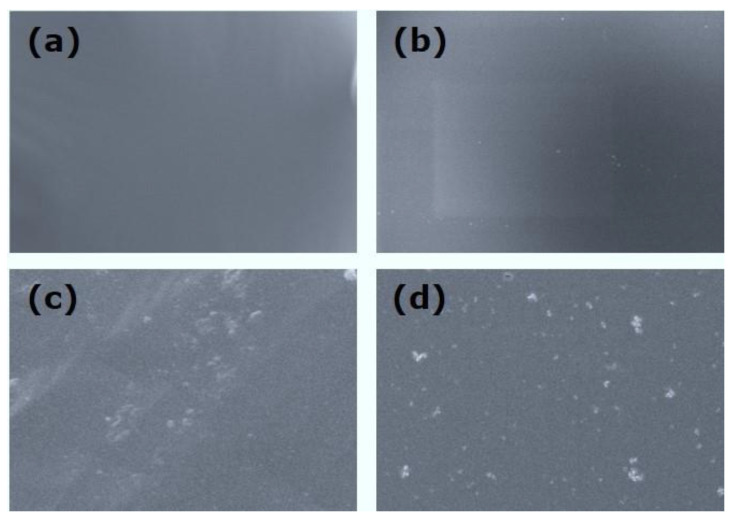
FE-SEM images of (**a**) PVA, (**b**) PVA/Pbz1, (**c**) PVA/Pbz3 and (**d**) PVA/Pbz5.

The morphology of the polybenzoxazine and PVA/Pbz composite films was analyzed by SEM analysis and the figure (Figure 5) displays the SEM images of Pbz and PVA/Pbz films. As could be observed, the surface of Pbz is smooth and even (Figure 5a), whereas PVA/Pbz films show roughened and irregular structures. The dispersion of the polymer particles could be clearly seen in the SEM images. The dispersion is uniform without any agglomeration in PVA/Pbz1 (Figure 5b), whereas few agglomerations could be found in some parts of PVA/Pbz3 and PVA/Pbz5.

### 3.5. TGA Analysis

**Figure 6:**
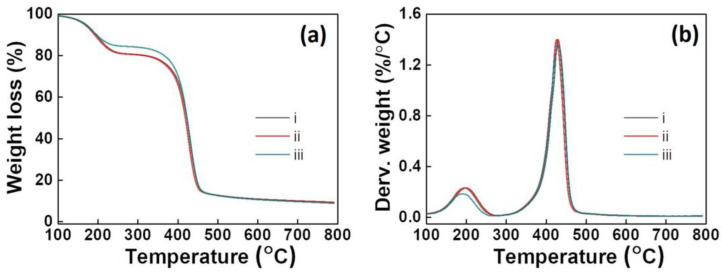
(**a**) TGA and (**b**) DTG graphs of (i) PVA/Pbz1, (ii) PVA/Pbz3 and (iii) PVA/Pbz5.

The thermal stability of the PVA/Pbz composite films was analyzed by TGA analysis. Figure 6a,b shows the TGA curves and DTG curves of the composite films. All three films show a similar degradation behavior, exhibiting a two-step degradation process. Generally, the degradation process of PVA starts at 200 °C. The PVA/Pbz films show the first degradation process between 175 and 250 °C, whereas the second degradation process starts at 350 °C, corresponding to the maximum degradation. The hydrogen bonding interactions between PVA and Pbz extend the degradation process up to 350 °C, after which the film loses its stability. The char yield obtained at 800 °C was found to be 10%. As the amount of Pbz is very low (max. 5 wt. %) in the whole matrix, a smaller amount of char remains at 800 °C.

### 3.6. Swelling Behavior of the Composite Film

**Figure 7:**
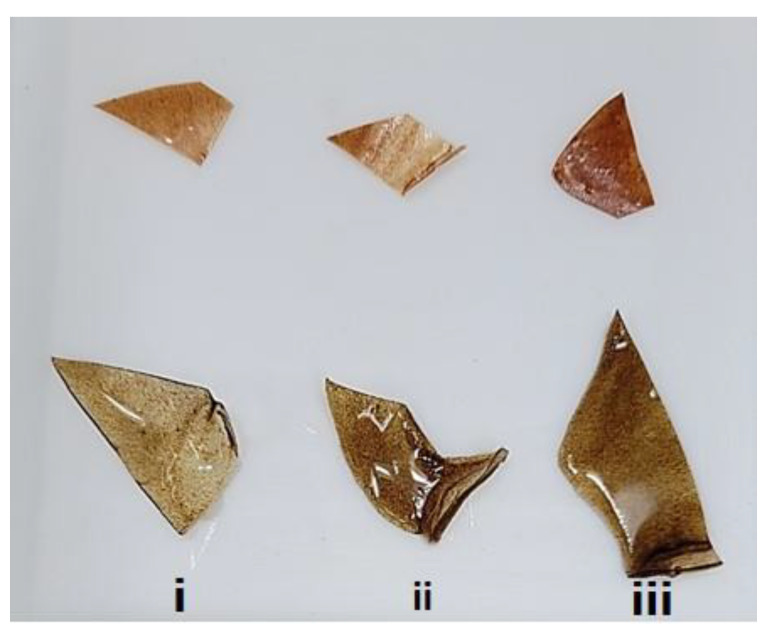
Images of the composite films (**top**) and swelled gels (**bottom**) [(**i**) PVA/Pbz1, (**ii**) PVA/Pbz3, and (**iii**) PVA/Pbz5].

The swelling behavior of the PVA/Pbz composite films was analyzed in a 1 M KOH solution. To start with, a small piece of the film was cut, and its weight was measured. This was then immersed in a 1 M KOH solution for 1 h. After this, the gel was taken out and the excess KOH solution was wiped softly with a tissue paper. Care was taken not to damage the gel. The weight of the gel was noted. The swelling % of the PVA/Pbz gels was calculated using the formula given below.

(2) $$\mathrm{Swelling}\;(\%)=W_{s}-W_{f}/W_{f}\times 100$$

where W_s_ is the weight of the swelled gel and W_f_ is the weight of the polymer film. As could be observed from Table 1, the swelling % of the gel was calculated to be 100.44% for PVA/Pbz1, 133.72% for PVA/Pbz3 and 153.47% for PVA/Pbz5. The swelling ratio of PVA/Pbz5 is higher than the other two, which suggests that, in PVA/Pbz5, there are more hydroxyl groups from both PVA and Pbz (phenolic hydroxyl) that could interact with additional water molecules and thus increase the swelling. The images of PVA/Pbz composite films and gels are displayed in Figure 7. The swelled gels are denoted as PVA/PbzG1, PVA/PbzG3 and PVA/PbzG5.

### 3.7. Tensile Property of the Composite Film

**Figure 8:**
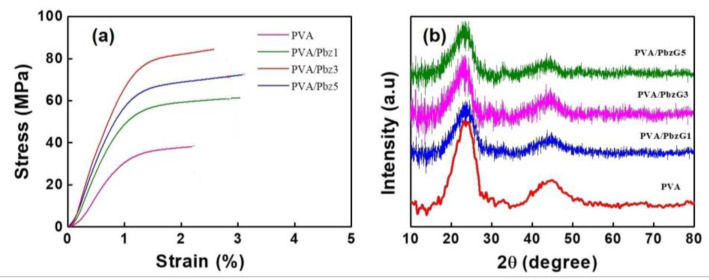
(**a**) Tensile measurements and (**b**) XRD graphs of PVA and PVA/Pbz films.

The mechanical properties of Polyvinyl Alcohol (PVA) and PVA/Pbz drawn fibers are elucidated in Figure 8a, showcasing distinctive stress–strain curves and average mechanical characteristics for both materials. These curves unveil notable disparities between the pure PVA fibers and their composite counterparts. The composite fibers display a markedly steeper initial slope on the stress–strain curve in contrast to pure PVA, indicating a heightened Young’s modulus, which is a measure of stiffness. The incorporation of Pbz substantially augments stiffness, as evidenced by an increase in values from 37 MPa for pure PVA to 82 MPa for the composite containing 5 wt% Pbz. Moreover, the tensile strength, denoting the maximum stress a fiber can endure before fracturing, exhibits a significant enhancement in composite fibers compared to pure PVA. Particularly noteworthy is the nearly twofold increase in tensile strength observed in PVA/PbzG5 composites compared to their pure PVA counterparts. Nevertheless, there exists a trade-off with higher Pbz content. The tensile strength experiences a notable decline as the Pbz content within the PVA/Pbz composite (referred to as PVA/PbzG5) is further escalated. This decline can be attributed to various factors pertaining to the composite’s structure and properties: Inadequate dispersion of Pbz particles within the PVA matrix, resulting in the formation of weak regions within the composite structure. Feeble interaction between PVA and Pbz, which is pivotal for ensuring efficient stress transfer. A weak interface between the two materials may impede the utilization of the Pbz’s reinforcing capabilities. Insufficient alignment of PVA and Pbz polymer chains during the fiber drawing process, thereby limiting the overall strength of the composite.

### 3.8. XRD Analysis

Examining the X-ray powder diffraction profile (XRD) depicted in Figure 8b provides insights into the crystalline arrangement within the melt-crystallized PVA film. The presence of sharp peaks signifies the crystalline nature of the material, with a prominent peak observed at 2θ = 22° accompanied by a shoulder at 2θ = 24°. These characteristics are indicative of atactic PVA crystallinity, wherein the polymer chains lack a regular helical structure. The diffraction pattern suggests a trans-planar conformation for the polymer chains within the PVA crystal lattice, as depicted in Figure 8b. This conformation implies that the polymer chains are stretched out and aligned in a planar fashion. Estimations based on peak intensities suggest a degree of crystallinity of approximately 65%. However, further investigation is warranted to ascertain the precise dimensions of the crystalline domains. Figure 8b illustrates the XRD profiles of the PVA/Pbz hydrogel samples post-background subtraction. Additionally, the diffraction patterns of carbon, a principal constituent of these hydrogels, are provided for reference. The hydrogel profiles exhibit two broad halos centered at 2θ ≈ 24° and 42°, corresponding to characteristic peaks of carbon. Furthermore, a subtle peak around 2θ = 21° indicates the presence of minor crystalline PVA aggregates within the hydrogel network.

### 3.9. Performance of the SC Device

A two-electrode system was used to analyze the performance of the SC device fabricated with the swelled gels. PVA/PbzG5 was chosen for the electrochemical measurements, due to its higher swelling ratio. The PVA swelled gel was taken for comparison analysis. The fabrication of the two-electrode system was performed in several steps. Firstly, the activated carbon (AC) was mixed with PVDF (ratio of AC and PVDF is 95:5), and a few drops of NMP was added to form a slurry. This slurry was then coated onto the Ni foam, leaving some space for the connection. This forms the electrode part. In a similar way, another electrode part was made with AC. The prepared PVA/PbzG5 was sandwiched between the two-AC electrode, forming an AC//PVA/PbzG5//AC symmetric supercapacitor device. The whole setup was wrapped with cellophane tape to form a complete device.

**Figure 9:**
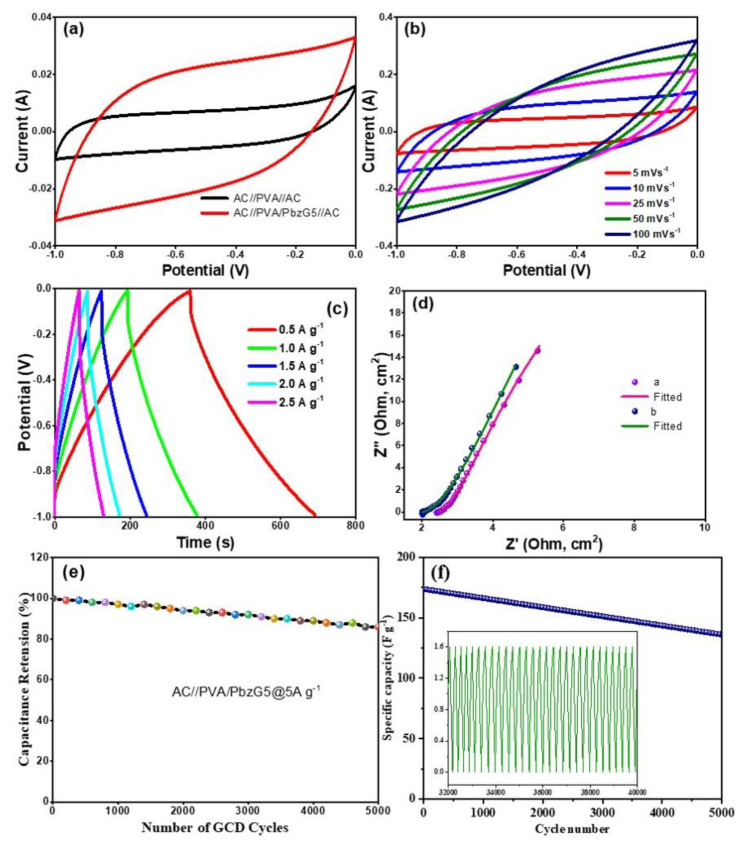
(**a**) CV curves of symmetric SCs at 50 mV s^−1^, (**b**) CV, (**c**) GCD, (**d**) EIS spectra, (**e**) cyclic stability and (**f**) capacitance retention of AC//PVA/PbzG5//AC.

Figure 9a displays the CV curves of symmetric SCs fabricated with PVA and PVA/PbzG5, at a scan rate of 50 mV s^−1^. Both the SCs show EDLC behavior (electrical double-layer capacitance) in the negative potential range (−1.0 to 0 V). As could be seen, the area within the curve is higher for PVA/PbzG5 when compared with PVA. This could be due to the fact that the network formed between PVA and Pbz enables it to store more charge than the PVA alone. Figure 9b shows the CV curves of AC//PVA/PbzG5//AC at different scan rates from 5 to 100 mV s^−1^. The area of the CV curve is increased with increasing scan rates. Figure 9c depicts the GCD curve of AC//PVA/PbzG5//AC at different current densities from 0.5 to 2.5 A g^−1^. The specific capacitance calculated from equation [2] was found to be 174.0 F g^−1^ at 0.5 A g^−1^; 170.0 F g^−1^ at 1.0 A g^−1^; 160.5 F g^−1^ at 1.5 A g^−1^; 155.4 F g^−1^ at 2.0 A g^−1^; and 150.4 F g^−1^ at 2.5 A g^−1^. The EIS spectra in Figure 9d represents the impedance curves for AC//PVA/PbzG5//AC and AC//PVA//AC, showing a solution resistance of 2.1 and 2.5 Ω, respectively. The electrochemical results show that, by incorporating a smaller amount of Pbz into PVA, there is enhancement in the capacitance performance [32,33,34,35].

To evaluate the durability of the AC//PVA/PbzG5//AC electrode in regard to enduring repeated charging and discharging cycles, galvanostatic charge–discharge (GCD) was employed, at a current density of 0.5 A g^−1^. The GCD measurements demonstrated that the specific capacitance of the AC//PVA/PbzC5//AC electrode experienced a slight reduction, decreasing from 174 to 132 F g^−1^ after undergoing 5000 cycles. This outcome indicates a retention ratio of 76%, showcasing the electrode’s notable capacity to maintain performance over prolonged cycling periods, as depicted in Figure 9e. Additionally, the integral areas encompassed by the GCD curves during the initial and 5000th cycles at 0.5 A g^−1^, as presented in Figure 9f, further corroborate the exceptional cycling durability of the AC//PVA/PbzG5//AC electrode.

## 4. Conclusions

A gel polymer electrolyte containing both PVA segments and Pbz segments was fabricated by adopting a simple methodology: making use of self-curing polybenzoxazine chemistry. The brittleness of Pbz has been overcome by blending it with PVA. Free-standing films were produced due to the interactions between –OH groups of PVA, –OH and –NH_2_ groups of Pbz. DSC analysis showed that a processing temperature of 100 ℃ is sufficient to cure the benzoxazine monomer and to form PVA/Pbz films. The hydrophobicity of Pbz is greatly reduced by forming PVA/Pbz films (WCA of PVA/Pbz3 is 11.4°). The swelling percentage of PVA/Pbz5 composite film has increased to 153.47%. Moreover, the fabricated symmetric SC, AC//PVA/PbzG5//AC, showed improved capacitance performance, with a specific capacitance of 174 F g^−1^. The overall results show that a slight modification could bring about significant enhancement in energy storage applications.

## Figures and Tables

**Table 1:** Swelling percentage of PVA/Pbz composite films.

Sample	Film Weight (g)	Gel Weight (g)	Swelling (%)
PVA/Pbz1	0.0890	0.1784	100.44
PVA/Pbz3	0.0628	0.1468	133.72
PVA/Pbz5	0.1021	0.2588	153.47

## Data Availability

The data presented in this study are openly available in article.
